# The reporting of statistics in medical educational studies: an observational study

**DOI:** 10.1186/1471-2288-7-35

**Published:** 2007-07-20

**Authors:** Norman A Desbiens

**Affiliations:** 1 Department of Medicine, University of Tennessee College of Medicine, Chattanooga, USA

## Abstract

**Background:**

There is confusion in the medical literature as to whether statistics should be reported in survey studies that query an entire population, as is often done in educational studies. Our objective was to determine how often statistical tests have been reported in such articles in two prominent journals that publish these types of studies.

**Methods:**

For this observational study, we used electronic searching to identify all survey studies published in *Academic Medicine *and the *Journal of General Internal Medicine *in which an entire population was studied. We tallied whether inferential statistics were used and whether p-values were reported.

**Results:**

Eighty-four articles were found: 62 in *Academic Medicine *and 22 in the *Journal of General Internal Medicine*. Overall, 38 (45%) of the articles reported or stated that they calculated statistics: 35% in *Academic Medicine *and 73% in the *Journal of General Internal Medicine*.

**Conclusion:**

Educational enumeration surveys frequently report statistical tests. Until a better case can be made for doing so, a simple rule can be proffered to researchers. When studying an entire population (e.g., all program directors, all deans, and all medical schools) for factual information, do not perform statistical tests. Reporting percentages is sufficient and proper.

## Background

The inclusion of statistical tests has been inculcated into investigators as a necessary step in the reporting of medical research. While descriptive statistics may always be used (unless the data are too sparse), the employment of inferential statistics – statistics calculated from a study sample in order to make generalizations about a larger study target population – is not always appropriate.

One situation in which statistical testing must be used with caution is when tests are calculated from what may be a non-random sample [1]. In the medical education literature, this situation may occur when researchers use subjects from their own institutions. The calculation of inferential statistics in these studies is only justifiable if researchers can be assured that the findings from their institution are similar to the reference population. From a strict theoretical viewpoint, they should not be calculated in these studies unless the assumption is made that the institutional sample is similar to a random sample – an inference that cannot be proven from the study data. Readers must always make their own judgments about this matter.

Another situation in which inferential statistics should not be used is when the study targets the entire population [2]. This type of study is technically known as an enumeration and it occurs in medical educational research when surveys are sent to all medical schools or residency programs of a given type in the United States. In this type of complete enumeration study, there is no larger actual or theoretical population. For example, it would not make sense to say that the target population is all medical schools in the world or that there is a larger theoretical population of U.S. medical schools. A resident and I recently discovered a problem with an enumeration study in an article that I was helping her critically review in preparation for a journal club presentation. A study was therefore undertaken to discover how often inferential statistics are used for enumeration studies in the medical educational literature.

## Methods

For this observational study, we used PubMed to identify all articles (from 1987 to 2006) from two high impact factor journals – an educational journal (*Academic Medicine *N = 1096) and a general medical journal (*Journal of General Internal Medicine *N = 1204) that frequently publish educational studies – using the search term "survey." From this search, we identified all studies in which surveys were sent to an entire population of responders and determined whether inferential statistics that generate p-values, such as t-, Wilcoxon, Fisher's exact, and chi-square tests were used.

## Results

Eighty-four articles were found: 62 in *Academic Medicine *and 22 in the *Journal of General Internal Medicine*. Most studies surveyed responders at all U.S. or Canadian medical schools, all program directors or all clerkship directors about some aspects of the school or program.

Overall 38 (45%) of the articles reported or stated that they calculated statistics. Of these, 31 (86%) reported p-values, 19 (50%) reported that they used chi-square tests, 12 (32%) t-tests, 6 (16%) some type of multiple regression analysis that included p-values or confidence intervals, and 5 (13%) analysis of variance; 2 studies each calculated other statistics including Fisher's exact tests, Wilcoxon tests, Mann-Whitney tests, and confidence intervals; other statistics were used for 8 other studies.

*Academic Medicine *articles reported statistics in 22 of 40 articles (35%) while *Journal of General Internal Medicine *articles reported statistics in 16 out of 22 articles (73%). Statistics were more prevalent in more recent articles in *Academic Medicine*. For example, only 4 out of 30 (13%) before 1996 reported statistics compared to 18 out of 32 (56%) articles published afterwards.

## Discussion

This study demonstrates that statistics are frequently overused in articles from two prominent and highly-cited journals that report educational studies. In fact, since 1997 the majority of articles reporting enumeration studies have inappropriately included statistics.

The authors of one article that did not include statistics wrote directly to the issue in their methods section: "All analyses are based on the entire population of interest. Therefore, tests of statistical significance are not provided" [3]. In another article, the authors appeared conflicted by the issue: "These statistics are included for the interested reader but should be interpreted carefully, for the journals in this study do not, by one way of thinking, represent a sample but rather the entire population of interest" [4]. These authors should not have been stymied and should not have reported inferential statistics.

The findings of our study are worrisome for a number of reasons. The two journals that were studied are among the most prominent in their field and are highly cited. Authors may use them as role models in the reporting of educational studies. In addition, the concept of sampling is central to the whole of inferential statistics and is usually discussed in the early chapters of statistical textbooks [5]. If researchers are confused about a fundamental issue such as whether or not a group of subjects is a sample or an entire population, how are readers to be comforted that other more complex analytical issues have been validly addressed?

A reviewer of this article suggested that there may still be a role for statistics in finite populations by appealing to probability distributions that generated the scores in the population, and that statistical tests are appropriate to compare not the actual numbers in the population but the probability distributions that are imperfectly indicated by the values in the populations. One situation in which this might be the case is when a survey questions an entire population (deans, program or clerkship directors) about values, preferences or impressions such as is often done with Likert-type questions. In this case, the referent distribution might be envisioned to be the impressions of all persons who might hold the office of the person who is responding to the questionnaire. This consideration would not be germane for factual information often queried about in surveys. Dusoir has suggested that "statistics is a collection of warring factions, with deep disagreements over fundamentals" and differences in reporting statistics from finite probabilities may be one of these fundamental issues [6]. On the other hand, Oakes may be correct that "many researchers retain an infatuation with statistical tests" [7].

In addition to confusion about fundamental issues in statistics, the increasing prevalence of statistics in these studies over time suggests that the inappropriate use of statistical packages may be partly to blame. Many of the studies included statements to the effect that data were entered in statistical packages, when for all of these studies a spreadsheet program would have been more than adequate. While statistical packages can generate tests quite readily, the proper interpretation of their output is the responsibility of the investigator. Anthony has suggested that the "use of such (packages) does, unfortunately, also allow you to perform meaningless statistics and incorrect statistical tests, and give misleading or wrong interpretations" [8].

This study did not sample all articles in the medical literature that have reported enumeration studies. However, it reports on all such studies that have been published in two leading journals that report medical educational studies. We suspect that this problem is also rampant in other journals that report this type of study. The proband case that led to this study was published in another leading journal [9].

## Conclusion

The improper use of statistics in medical research has become a matter of much concern [10]. In an attempt to improve the situation, medical journals have begun taking a more prescriptive role in the research reports that they accept. Many now subscribe to the CONSORT statement for the reporting of randomized trials [11]. Other statements are being developed for other study designs [12,13]. Until a better case can be made, researchers can follow a simple rule. If they are studying an entire population (e.g., all program directors, all deans, all medical schools) and they are requesting factual information, then they do not need to perform statistical tests. Reporting percentages is sufficient and proper.

## Competing interests

The author declares that he has no competing interests.

## Authors' contributions

ND is the sole author.

## Pre-publication history

The pre-publication history for this paper can be accessed here:


